# Network pharmacology predicts targets and pathways of herbal components for the treatment of pneumonia: A review

**DOI:** 10.1097/MD.0000000000041372

**Published:** 2025-01-31

**Authors:** Dongxin Yang, Cuilian Chen, Qingshang Zhang, Jun Gong

**Affiliations:** a Central Laboratory of YunFu People’s Hospital, Yunfu, China; b YunFu Key Laboratory of Brain Diseases Research, Yunfu, China; c Department of Pharmacy, YunFu People’s Hospital, Yunfu, China.

**Keywords:** network pharmacology, pneumonia, signaling pathway, traditional Chinese medicine

## Abstract

Pneumonia is a respiratory disease with high pathogenicity and mortality. Traditional Chinese medicine (TCM) is a natural therapy that has proven effectiveness and safety. Although TCM has been found to be effective in treating pneumonia, further research is needed to determine the specific mechanism of action. This paper presents a literature search conducted in PubMed, Web of Science, and China National Knowledge Infrastructure (CNKI) databases using the keywords “pneumonia” and “network pharmacology.” After screening, we retained the literature related to TCM. The study found that, according to network pharmacology prediction, 4 types of TCMs–natural active compounds, single herb medicine, Chinese patent medicines, and multi-component herbal formulations–were effective in treating pneumonia. TCM components demonstrated a multi-target and multi-pathway approach to treat the disease. The diversity of targets and signaling pathways not only facilitates the investigation of TCM’s mechanism of action of TCM in pneumonia treatment but also offers novel insights and perspectives for innovative drug research and development.

## 1. Introduction

Pneumonia is defined as an inflammatory condition of the lungs that primarily affects the terminal airways, alveoli, and the interstitial tissue. This pathology is most often initiated by the presence of various pathogens, such as bacteria, viruses, and fungi. Moreover, pneumonia may also occur due to immune system impairments or as a consequence of pharmacological treatments.^[1]^ Despite the diversity of pathogens that cause pneumonia, the symptoms they produce are remarkably similar once the lungs are invaded. Consequently, the traditional Chinese medicine (TCM) approach of “dialectical diagnosis and treatment” offers distinct advantages.^[2]^ The consistent therapeutic success of TCMs in treating diseases continually inspires hope and astonishment, leading an increasing number of researchers to focus on uncovering the complex compositions and mechanisms of these medicines. However, the complexity of these components presents a challenge for understanding the interactions between TCMs and diseases.

**Figure 1.**
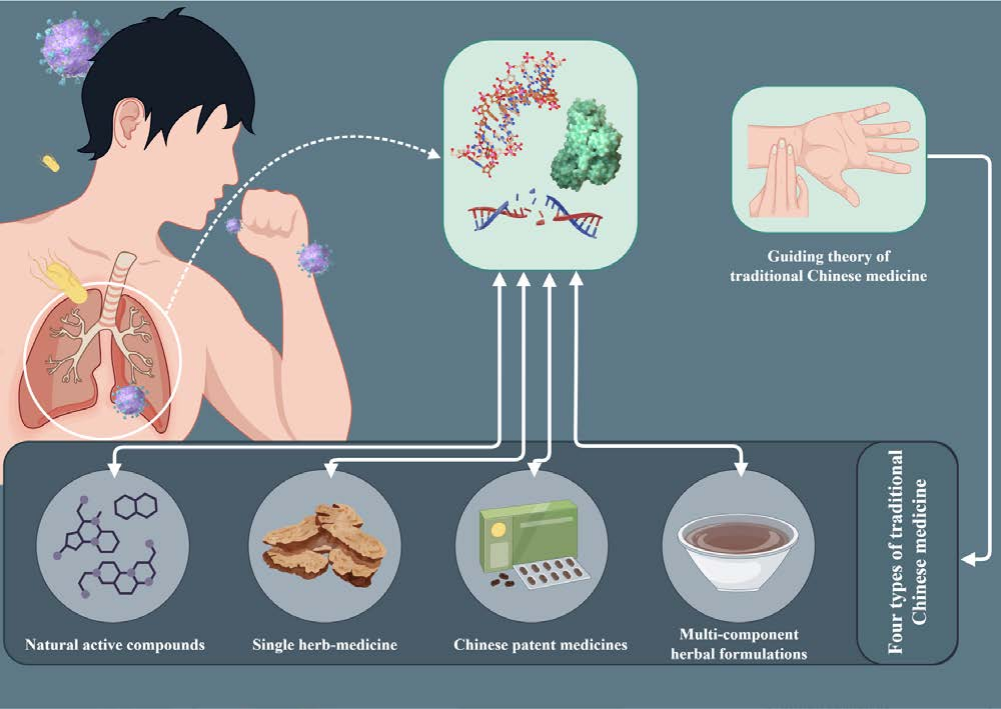
Therapeutic effects of 4 types of TCMs on pneumonia under the guidance of TCM theory.

Advancements in science and technology have ushered in the big data era of drug research, where network pharmacology (NP) plays a pivotal role in facilitating the research and development of drugs. This approach involves understanding the principles of drugs and diseases by exploring their mechanisms and pathways, which are now widely accepted. By integrating the TCM ingredient-target database with the disease gene database, NP enables the prediction of shared targets between TCM ingredients and pneumonia, thereby aiding researchers in narrowing their initial scope of study. The fusion of modern NP with TCM reveals the intricate network of TCM, which is characterized by multiple components, pathways, and targets. The quality and comprehensiveness of databases are critical limitations to the application and development of NP. Nevertheless, current NP techniques continue to provide valuable guidance and reference for researchers. A schematic of this review is presented in Figure 1.

This review aims to integrate the potential mechanisms of TCM components in the treatment of pneumonia, as predicted by NP methods, with a particular focus on those components already applied in clinical practice. Our goal is to provide new clinical interventions for pneumonia treatment, especially in the context of increasing antibiotic resistance, offering an effective alternative treatment option to improve patient outcomes and quality of life.

## 2. Methods

We employed a systematic approach for literature screening. Initially, a Medical Subject Headings (MeSH) keyword search was conducted, focusing on terms such as “pneumonia” and “network pharmacology.” A comprehensive literature search was then performed across multiple databases, including PubMed, Web of Science, and China National Knowledge Infrastructure, to identify relevant articles related to TCM. We systematically reviewed the search results to exclude irrelevant studies and removed duplicate records to ensure the uniqueness and reliability of the data. Ultimately, only those articles that met the inclusion criteria were retained for further analysis, thereby providing a solid foundation for the subsequent research.

## 3. Results

### 3.1. Natural active compounds

Employing contemporary medical techniques to isolate and identify active compounds represents an effective approach for investigating the material basis of the efficacy of TCM. Acquiring detailed information on the composition of TCMs is essential for modernizing this traditional practice. Concentrating specific compounds allows for a more convenient, intuitive, and precise analysis of the sources of efficacy in TCM. Moreover, this strategy aids in identifying the targets of the compounds.

Through NP and molecular docking, anemoside B4 was identified to have a mitigating effect on the release of several pro-inflammatory factors, including tumor necrosis factor (TNF)-α, interleukin (IL)-1β, and IL-6. Further studies using molecular docking and animal models confirmed the B4’s therapeutic potential against pneumonia induced by *Klebsiella pneumoniae* or influenza virus FM1 through modulation of the TLR4/Myd88 signaling pathway.^[3]^ Tianling Lou et al validated the predictions from network pharmacological results using a mouse model of pneumonia induced by *Pseudomonas aeruginosa*. They found that the aqueous extract of *T. hemsleyanum* leaves exerted an anti-inflammatory effect by maintaining Th17/Treg immune homeostasis, thus suppressing the inflammatory immune response.^[4]^ Gao et al identified a core network involving pro-inflammatory receptor signaling pathways, including TNF-α, IL-6, IL-1β, mitogen-activated protein kinase (MAPK), and RIG-I, as critical targets of the total flavones of *Abel-moschus manihot*.^[5]^ Total flavones of *Abel-moschus manihot* has been shown to protect the lungs by regulating the MAPK signaling pathway to expedite the clearance of Influenza A virus (IAV). A separate network pharmacological analysis indicated that pneumonia induced by IAV involves multiple signaling pathways, suggesting that total lignans enhance alveolar macrophage function and ameliorate lung injury in mice.^[6]^ The anti-influenza virus effects of the crude extract from the flowers of *T. chinensis* Bunge were attributed to the regulation of TLR3, IRF3, IFN-β, TAK1, and TBK1 expression in the TLR3 signaling pathway, which is believed to counteract influenza A (H1N1) virus.^[7]^ Huang et al reported that asiaticoside may be effective against coronavirus disease 19 (COVID-19) by targeting inflammatory and immune-related signaling pathways, showing a high affinity for core targets in molecular docking analyses.^[8]^ Moreover, a decrease in the expression of TLR3, IRF3, IFN-β, TAK1, and TBK1 was observed in response to H1N1. Shen et al used NP and molecular docking simulations to elucidate the mechanisms of action of F1012-2, a novel sesquiterpene lactone from *Eupatorium lindleyanum* DC., to treat viral pneumonia and identify EGFR, IL-1B, SRC, and CASP3 as promising targets for further development.^[9]^

Beyond the therapeutic potential of single compounds in treating pneumonia, the synergy between TCM compounds and western pharmaceuticals presents significant promise. Cui et al.’s NP study highlighted that the anti-influenza efficacy of Huangqin Su correlates strongly with the activation of signaling pathways, such as TLRs, MAPK, and TNF.^[10]^ The combination of Huangqin Su with oseltamivir phosphate enhances its effectiveness at reduced doses. Additionally, this pairing increased *Lactobacillus* abundance in the gut flora of the mouse models, potentially boosting the immune response. These findings offer valuable insights into clinical strategies for influenza treatment. Similarly, the combined application of vitamin C and glycyrrhizic acid activates the T-cell receptor signaling pathway, regulates Fc gamma R-mediated phagocytosis, and affects the ErbB and vascular endothelial growth factor signaling pathways. This dual action approach amplifies the immune response and mitigates inflammatory stress.^[11]^

### 3.2. Single herb-medicine

The term “single herb-medicine” does not denote a solitary component. TCM typically comprises multiple active ingredients, enabling a multifaceted modulation effect on diseases through various targets and pathways. Utilizing network pharmacological analysis of specific herbal medicines can identify high-quality compounds with significant developmental potential, greatly benefiting the advancement of new pharmaceuticals.

Zhang et al identified 5 significant targets of *Phlomis brevidentata* H.W. Li Radix involved in disease regulation, namely GAPDH, ALB, MAPK 1, AKT 1, and EGFR, through network pharmacological analysis.^[12]^ Radix Isatidis (Banlangen), widely recognized for its anti-inflammatory and immune response modulation properties, acts on targets such as PTGS1, PTGS2, and NOS2 and regulates signaling pathways including RAGE, IL-17, and TNF, thereby effectively combating COVID-19.^[13]^ Wang et al identified 9 common targets between almonds (Xingren) and COVID-19.^[14]^ Molecular docking showed that licochalcone B had the best binding energy with PTGS2 (−9.33 kJ/mol), indicating its potential for further development. According to Li et al, Radix Salviae Miltiorrhizae is likely a target of PTSG2 based on NP predictions, potentially playing a regulatory role in radiation pneumonia through the PI3K-AKT, HIF-1, and TNF signaling pathways, which is beneficial for multi-target resistance.^[15]^ *Houttuynia cordata* Thumb (*H. cordata*; Saururaceae) involves 4 signaling pathways (PI3K-Akt, Jak-STAT, MAPK, and NF-κB). Molecular docking and dynamics simulations confirmed stable binding between 4 metabolites (Afzelin, Apigenin, Kaempferol, and Quercetin) and 6 targets (DPP4, ELANE, HSP90AA1, IL-6, MAPK1, and SERPINE1) that were also reported to bind ACE2 and 3CLpro of SARS coronavirus 2, laying a theoretical foundation for further research on *Houttuynia cordata* Thumb’s effective components and action mechanism in pneumonia treatment.^[16]^ Methyl 4-prenylcinnamate, tormentic acid, and eugenol in *Lithospermum erythrorhizon* demonstrated potential binding to their targets RELA, TNF, and VEGFA, aiding in the inhibition of cellular inflammation via deactivation of the MAPK signaling pathway deactivation, also being explored for COVID-19 potential.^[17]^ *Achillea millefolium* (Yarrow) can inhibit or downregulate Cyclooxygenase II, suggesting its anti-inflammatory properties. According to Tilwani et al, Yarrow exhibits a significant inhibitory potential.^[18]^ NP highlighted the crucial roles of the TLR4/NF-κB signaling pathway in the anti-inflammatory and antioxidant effects of “*Pterocephalodes hookeri* and *Onosma hookeri*” (“*P-O*”) combination therapy.^[19]^

The combination of these 2 herbs often synergizes and contributes to their own regulatory targets for the same disease. Astragali Radix (AR) and Atractylodis Macrocephalae Rhizoma (AMR) are widely used medicinal herbs. Core targets, such as TNF, IL-6, IFNG, IL-1B, IL-10, IL-4, and TLR9, were identified through NP analysis. Synergistic components effective in treating pneumonia have been isolated from AR ((3R)-3-(2-hydroxy-3,4-dimethoxyphenyl)-7-chromanol, formononetin, quercetin, and kaempferol) and AMR (atractylone, 14-acetyl-12-senecioyl-2E, 8E, 10E-atractylentriol, and α-amyrin). Therapeutic strategies for pneumonia include immune diseases, infectious diseases, and organismal diseases.^[20]^ In the realm of TCM dietary culture, AR and AMR are deemed dual-purpose medicinal herbs, indicating their preventive use in daily life to fortify the body’s defenses against early stages of diseases. This underscores traditional Chinese medical theory’s focus on disease prevention.

### 3.3. Chinese patent medicines

Modern pharmaceutical production technology has revolutionized the manufacturing. The unique flavors of herbs frequently result in low patient compliance. Transforming these herbs into Chinese patent medicines can effectively mask their flavor, while also offering advantages such as extended shelf life, precise dosing, and enhanced ease of transport and preservation.^[21]^

During the COVID-19 pandemic, Xuebijing (XBJ) injections have been extensively employed and have demonstrated effectiveness against severe viral infections or inflammatory conditions. XBJ targets significant molecules, such as ACE2 and AKT1, blocking viral cell entry, replication, and dissemination, thus manifesting its antiviral properties.^[22]^ Additionally, XBJ is capable of reducing the levels of inflammatory mediators, including IL-2, IL-4, and TNF-α, and influences various signaling pathways, including PI3K-AKT, NF-κB, MAPK, and Toll-like receptors.^[23]^ XBJ may offer a therapeutic benefit for acute respiratory distress syndrome through the modulation of immune cell/cytokine pathways and mitigation of cytokine storms. Notably, TNF-α was directly and indirectly suppressed by XBJ, whereas IL-6 was mainly inhibited indirectly by XBJ.^[24]^

Shen Qi Wan may regulate various biological functions and signaling pathways relevant to COVID-19 by directly or indirectly binding kaempferol, quercetin, and luteolin to ACE2, 3CLpro, PLpro, and PTGS2.^[25]^ Wang et al proposed through NP and cellular experiments, Wang et al proposed that Chaishi Tuire Granules could inhibit inflammatory responses by affecting the TRAF6/MAPK14 signaling pathway.^[26]^ Chaiyin particles can suppress the release of inflammatory factors such as IL-6 and TNF-α through the AGE-E2 signaling pathway, playing a preventive role against COVID-19 by blocking ACE2 with fortunellin and baicalin.^[27]^ The therapeutic efficacy of Lianhua Qingwen (LHQW) capsules in treating viral pneumonia might be linked to their modulation of the TLR4/NF-κB signaling pathway in the lungs, in addition to restoring intestinal flora and gut barrier function, aligning with the TCM principle that “the lung and large intestine are interconnected.”^[28]^ Su et al discovered that components of LHQW, including isochlorogenic acid B, isoforsythiaside, forsythoside E, rutin, quercitrin, and hesperidin, target crucial biological molecules, such as 3CL, ACE2, COX2, HA, IL-6, and NA, interfering with signaling pathways, such as IL-17, T cell receptor, Th17 cell differentiation, TNF, Toll-like receptor, MAPK, and apoptosis, thus offering anti-inflammatory, antiviral, and immune-regulatory benefits.^[29]^ Additionally, LHQW blocks the p53-mediated intrinsic apoptosis pathway to mitigate lipopolysaccharide (LPS)-induced acute lung injury.^[30]^

Table 1 lists the various Chinese patent medicines extensively utilized throughout the COVID-19 pandemic, accompanied by a broad range of proprietary TCMs that are effective in treating pneumonia.^[31–48]^

**Table 1:** Names and mechanisms of commonly used Chinese patent medicines

Serial number	Name of Chinese patent medicine	Mechanism	References
1	Feilike mixture	Core targets: TNF, AKT1, IL-6, VEGFA, and MAPK3. Core compositions: resveratrol, stigmasterol, beta-sitosterol, sesamin, and quercetin.	^[31]^
2	Re-yan-ning mixture	Cell proliferation and survival are mainly regulated by IL-6/IL-10/IL-17, Bax/Bcl-2, COX-1/COX-2, NF-κB, and TNF-α signaling pathways.	^[32]^
3	Jingfang granule	By inhibiting the STAT3/IL-17/NF-κB pathway to reduce the inflammatory response caused by Pseudomonas aeruginosa acute lung infection.	^[33]^
4	Compound Kushen injection	Blocks viral replication and its binding to SARS-CoV-2 and ACE2 receptors. Regulates IL-6-mediated signaling pathway, TNF signaling pathway, and steroid hormone biosynthesis.	^[34]^
5	Jinchan Oral Liquid	Treatment of respiratory syncytial virus pneumonia mainly through TNF signaling pathway and IL-17 signaling pathway.	^[35]^
6	Reduning injection	Regulate ACE2, Mpro, and papain-like protease. Regulates cytokine levels and inflammation, and antipyretic activity by modulating the expression of MAPKs, PKC, and p65 nuclear factor NF-κB.	^[36]^
7	Modified Xiebai Powder combined with azithromycin	Possible treatment of pneumonia through signaling pathways such as IL-17.	^[37]^
8	YuPingFeng Powder or YuPingFeng Granules	Important Ingredients: Wogonin, prim-*O*-glucosylcimifugin, 5-*O*-methylvisamminol, astragaloside Ⅳ and 5-*O*- methylvisamminol (hydroxylation). Core targets: RELA, TNF, IL-6, MAPK14, and MAPK8.	^[38]^
		Core genes: IL-1B, IL-6, CXCL8, TNF, and IL-10. Mainly involved in the body’s immune-inflammatory response and inhibition of cell migration.	^[39]^
9	Jiu Wei Zhu Huang San	Potential therapeutic targets: TNF, IL-6, IL-1B, IL-2, JUN, MAPK1, MAPK8, and EGFR.	^[40]^
10	Qiangli Wuhu mixture	Strong protective effect against LPS-induced pneumonia in mice by inhibiting TLR4/NF-JB/NLRP3.	^[41]^
11	Qinbaiqingfei concentrated pills	Inhibit the NF-κB signaling pathway through SIRT 1, IL-10, MMP 9, CTNNB 1, EGFR and other targets to play the role of immune regulation, metabolism regulation, and disease treatment.	^[42]^
12	Qinbaohong Zhike oral liquid	Attenuating LPS-induced lung tissue injury and inflammatory response in young rats by inhibiting OLFM 4 expression.	^[43]^
13	Qingfei Xiaoyan Wan	Regulates PI3K/AKT and Ras/MAPK pathways to effectively inhibit pathogenic bacterial infection.	^[44]^
14	ShuFeng JieDu capsule	Core genes: RELA, MAPK1, MAPK14, CASP3, CASP8, and IL-6.	^[45]^
15	Qing-Fei-Da-Yuan granules	Regulates genes co-expressed with ACE2. Regulates inflammatory and immune-related signaling pathways. Affects COVID-19 3CL hydrolase and ACE2 binding-related capacity.	^[46]^
16	Xiyanping injection	Inhibits MAPK, TNF and other intracellular signaling pathways to inhibit the release of multiple inflammatory factors. Acts on protein targets such as HSP90AA1.	^[47]^
17	Xiebai San	Inhibits MyD 88/NF-κB p65-mediated inflammatory signaling. Inhibits PI3K/Akt-mediated apoptosis.	^[48]^

### 3.4. Multi-component herbal formulations

Compound decoctions represent a traditional dosage form within the theoretical framework of TCM, characterized by the combination of various herbs in precise proportions to enhance their therapeutic effects. This technique of blending herbs into compound preparations signifies a remarkable innovation in TCM, showcasing the profound ingenuity of the ancient Chinese people. The imperative to explore the material basis underlying the efficacy of these compounded TCM preparations has arisen with the advent of modernization. However, the intricate composition of TCM compounds poses challenges for further investigation. Currently, NP offer new perspectives and avenues for researching TCM compounding by initially forecasting the targets of multiple components.

Yi et al analyzed the Ruhao Dashi formula (RDF) using NP and discovered that it modulates relevant targets via the MAPK, TNF, PI3K-Akt, and IL-17 signaling pathways. RDF’s multiple active ingredients of RDF exhibited a stronger binding affinity to targets linked with acute pneumonia than aspirin.^[49]^ Baicalein and quercetin in the Huashi Baidu formula displayed therapeutic effects against COVID-19 by modulating various signaling pathways, including ACE2.^[50]^ Mongolian medicine has emerged as a potential remedy for pulmonary infectious diseases, with NP analysis of core group medicines showing Mongolian medicine’s effect on pulmonary infectious diseases by regulating the AGE-RAGE, IL-17, and TNF signaling pathways by targeting VEGFA, IL-6, TP53, and AKT1, among others.^[51]^ Qian Liu and colleagues investigated Feiduqing through NP analysis and found that its components reduced the levels of PGE2, TNF-α, cAMP, IL-1β, and MPO while enhancing the anti-inflammatory factor IL-10 content. These findings complement the pharmacological results of animal experiments.^[52]^ Huang Lian Jie Du decoction, widely used in COVID-19 treatment, may inhibit the overproduction of inflammatory factors via the TNF, NF-κB, and HIF-1 signaling pathways, potentially safeguarding COVID-19 the organs.^[53]^ Ma Xing Shi Gan decoction demonstrated inhibitory effects on early SARS coronavirus 2 invasion and anti-inflammatory and immune regulatory effects through the TLR signaling pathway, aiding in cytokine storm prevention.^[54]^ NP and molecular docking analysis showed the strong affinity of quercetin and wogonin with CXCL8, CCL2, or IL-1B. In vitro experiments confirmed their ability to mitigate the inhibition of proliferation and apoptosis in LPS-induced A549 cells.^[55]^ Research by Zhang et al on Shiwei Qingwen decoction indicated that it could alleviate the inflammatory response in acute lung injury by blocking TLR4/NF-κB and NLRP3 inflammasome activation.^[56]^ Xu et al suggested that Yinlai decoction combats pneumonia from pathogens by modifying the pulmonary and systemic environments, with the regulation of the inflammatory factor IL-6 being a potential key target.^[57]^

In pneumonia research, MAPK is a commonly targeted protein that is often included in NP analyses of various herbal formulas. The Xuanbai-Chengqi decoction targets both MAPK and TNF, thereby inhibiting the release of inflammatory cytokines. Xuanbai-Chengqi reduces the protein expression of TLR7, MyD88, and p-NF-κB65, contributing to intestinal homeostasis regulation and anti-inflammatory properties. It is also used to treat lung and intestinal damage, reinforcing the TCM principle that “the lung and large intestine are interconnected.”^[58]^ Research by Ji et al posits that the Qinggan Yin formula may exert anti-inflammatory effects through the MAPK signaling pathway and oxidative stress pathway.^[59]^ Acute pneumonia, a seasonal inflammatory disease, responds well to RDF, which modulates the MAPK, TNF, PI3K-Akt, and IL-17 signaling pathways.^[49]^ Further, a study by Guo et al on the Xijiao Dihuang decoction combined with Yinqiao powder revealed a close association between multiple pathways within the MAPK signaling pathway and identified key differentially expressed targets (EGFR, FOS, MAPK1, MAP2K1, HRAS, NRAS, and RELA) between influenza virus-infected and uninfected individuals.^[60]^ This suggests that these targets play a significant role in treating influenza virus-induced pneumonia with the Xijiao Dihuang decoction combined with Yinqiao powder.

## 4. Discussion

The application of NP technology to investigate the correlation between TCM components and disease targets is a prime example of the modernization of TCM. The emergence and utilization of NP technology have facilitated the convenient verification of drug components and disease targets at a theoretical level, effectively bridging the gap between efficacy prediction and drug development. The practical value of NP is undeniable, and it is important to acknowledge their limitations.

The current NP research landscape exhibits a notable degree of homogeneity. A predominant proportion of contemporary articles in this field adhere to a standardized research methodology, thereby promoting consistency in the research process. However, this uniformity often comes at the expense of distinct study characteristics and a comprehensive experimental validation. Furthermore, certain compounds, such as sophoretin and β-sitosterol, have garnered considerable attention across various cyberpharmacology studies because of frequent predictions or purported high targeting potential.^[61]^ This phenomenon may have resulted in an undue emphasis on specific substances, detracting from a more holistic exploration of diverse compounds. The robustness of network pharmacological studies depends heavily on the quantity and quality of available data. Regrettably, the relatively short development time of databases pertaining to TCM and the intricate composition of TCM remedies pose significant challenges for the establishment of comprehensive databases. When employing NP to investigate TCM, the focus is typically on identifying the active constituents within these remedies. However, this approach deviates from the principles of TCM, which advocate for a holistic perspective encompassing the concepts of “monarch, minister, assistant, and guide.” Within this framework, medicinal agents are viewed as interdependent entities that complement and regulate each other, guided by the principle of tailoring treatments based on specific patterns of disharmony. Presently, pharmacological exploration of Chinese herbal medicine within the NP domain remains rudimentary, primarily restricted to basic investigations centered on a limited repertoire of known ingredients. Compounded by various constraints, including methodological limitations and resource constraints, the resulting research outcomes often exhibit a high degree of similarity, underscoring the urgent need for more diverse and expansive datasets. Moreover, the application of TCM theory within the context of NP remains challenging, as it necessitates a nuanced understanding of dosage considerations and differentiation of target populations based on traditional Chinese medical principles.

Contradiction serves as an inherent catalyst for the advancement of various endeavors, and the constraints within the realm of NP in TCM serve as potent stimuli for its growth and refinement. It is imperative to expand and deepen research into Chinese medicinal practices, thereby providing ample resources and establishing a robust data platform for the exploration of NP within this domain. Building upon this foundation, the incorporation and fusion of multidisciplinary resources, along with the development of innovative research methodologies, can enhance the breadth and standardization of investigations. Research endeavors focusing on Chinese medicinal practices should strive to align with the principles of TCM whenever feasible. For instance, the Traditional Chinese Medicine Systems Pharmacology Database and Analysis Platform database frequently employ parameters, such as oral bioavailability and drug-likeness, for data screening.^[62]^ According to TCM theory, the fundamental attributes of TCM encompass the “four qi and five flavors,” as well as considerations regarding elevation, levitation, sinking, and meridian affiliations. Augmenting the Traditional Chinese Medicine Systems Pharmacology Database and Analysis Platform database with labels and screening criteria reflecting these characteristics of Chinese medicines can bring the study of NP closer to TCM principles. Simultaneously, this enhancement can facilitate a better alignment of Chinese medicinal treatments with the specific needs of individuals.

## 5. Conclusions

TCM represents the wisdom of the Chinese people and is an outstanding representative of China’s cultural heritage. It provides wisdom across time and space and is regarded as a valuable asset for mankind. This study offers a new perspective on the active targets and mechanisms of Chinese medicinal ingredients utilizing NP, focusing on the following: natural active compounds, single herb medicine, Chinese patent medicines, and multi-component herbal formulations. TCM, whether employed as an individual agent or in synergistic combinations, exhibits multi-target and multi-pathway therapeutic effects against pneumonia. This multiplicity underpins TCM’s therapeutic potency. This research elucidates a comprehensive set of targets and signaling pathways, providing strategic and theoretical support for continued investigation into TCM’s role of TCM in the treatment of pneumonia.

## Acknowledgments

We greatly appreciate the financial support from the Guangdong Province Bureau of Traditional Chinese Medicine Scientific Research Subject (Grant No. 20241398), and the Research Foundation of YunFu People’s Hospital (Grant No. A20232001).

## Author contributions

**Conceptualization:** Dongxin Yang.

**Data curation:** Dongxin Yang, Cuilian Chen, Qingshang Zhang.

**Formal analysis:** Dongxin Yang.

**Software:** Dongxin Yang.

**Writing – original draft:** Dongxin Yang, Cuilian Chen.

**Writing – review & editing:** Qingshang Zhang, Jun Gong.
